# The lingering legacy of one letter in antimicrobial susceptibility testing

**DOI:** 10.1099/jmm.0.002082

**Published:** 2025-10-07

**Authors:** Saied Ali, Fidelma Fitzpatrick

**Affiliations:** 1 Department of Clinical Microbiology, Royal College of Surgeons in Ireland, Dublin, Ireland; 2 Department of Clinical Microbiology, Beaumont Hospital, Dublin, Ireland

**Keywords:** antimicrobial stewardship, antimicrobial susceptibility testing, dose optimization, European Committee on Antimicrobial Susceptibility Testing (EUCAST), intermediate category, susceptible increased exposure

## Abstract

For decades, the ‘Intermediate’ (I) category in antimicrobial susceptibility testing was frequently misinterpreted as indicative of therapeutic failure, rather than an opportunity for dose optimization. This misunderstanding shaped prescribing behaviour, antimicrobial trial design and guideline development. The 2019 European Committee on Antimicrobial Susceptibility Testing redefinition of the ‘I’ category as ‘Susceptible, Increased Exposure’ (SIE) highlighted the potential for treatment efficacy through dose adjustment, challenging entrenched prescribing behaviours and exposing limitations in historical trials, guidelines and surveillance practices. Antimicrobial stewardship that employs SIE-based strategies can preserve narrower-spectrum agents. However, the misapplication of ‘I’ can promote unnecessary broad-spectrum antimicrobial prescribing. Surveillance systems, clinical decision support and antimicrobial stewardship should be continuously updated to reflect current pharmacological principles and thereby enhance patient care outcomes.

## Introduction: misunderstanding the ‘I’

For decades, the ‘I’ category in antimicrobial susceptibility testing occupied an ambiguous space between ‘Susceptible’ and ‘Resistant’. Initially intended to reflect clinical uncertainty, ‘Intermediate’ was widely misinterpreted as a proxy of likely therapeutic failure rather than as a prompt to consider alternative dosing strategies [1]. This misinterpretation profoundly shaped antimicrobial prescribing behaviours, guided escalation decisions and influenced clinical trial design, surveillance data interpretation and guideline development.

In response, in 2019, the European Committee on Antimicrobial Susceptibility Testing (EUCAST) revised the definition of ‘I’ to ‘Susceptible, Increased Exposure’ (SIE) or ‘Susceptible, Dose Dependent’ [1]. This reclassification emphasized that clinical success is attainable when higher or optimized dosing regimens are employed, reflecting modern pharmacokinetic/pharmacodynamic (PK/PD) principles rather than rigid binary thresholds [2]. However, this conceptual shift also highlighted the fragility of the evidence base underpinning many treatment recommendations, many of which were built on studies that excluded ‘I’ isolates or grouped them with resistant strains. As a result, the true clinical efficacy of several antimicrobial agents, particularly *β*-lactams, may have been underestimated.

Historically, the ‘I’ susceptibility category was rarely viewed as actionable and therefore of limited value in guiding antimicrobial therapy. In practice, clinicians often avoided using ‘I’-classified agents, especially in severe infections, opting instead for broader-spectrum or newer alternatives [2,3]. This conservative prescribing pattern, shaped by the misinterpretation of the ‘I’, not only distorted perceptions of antimicrobial efficacy but also contributed to the unnecessary escalation of therapy. The redefinition of ‘I’ by EUCAST compels a fundamental reassessment: had dose optimization been systematically applied in pivotal trials, how many ‘I’ isolates might have exhibited clinical susceptibility? And how many treatment escalations to carbapenems or novel agents could have been avoided?

By reframing ‘I’ to reflect dose-dependent susceptibility, EUCAST has challenged entrenched prescribing behaviours and highlighted the importance of integrating PK/PD considerations into both research and clinical practice. This paradigm shift underscores the need to revisit historical datasets and treatment paradigms to ensure that future decisions are grounded in evidence reflecting current pharmacological understanding, ultimately promoting more judicious and effective antimicrobial use.

## Real-world impact: lessons from *Pseudomonas aeruginosa*

EUCAST’s 2019 updates substantially reshaped susceptibility profiles for *P. aeruginosa*. Most *β*-lactams, such as piperacillin–tazobactam and ceftazidime, were reclassified from ‘S’ to ‘I’, not due to resistance mechanisms, but because standard doses were insufficient without dose optimization. Meropenem remained ‘S’, making it an apparently safe choice.

This shift produced unintended consequences. A Swiss study reported a tenfold increase in meropenem prescriptions for *P. aeruginosa* from 3.4 to 30.2%, attributable to its continued ‘S’ classification under the new definitions [4]. Similarly, a multicentre French study reported that overtreatment with broad-spectrum agents, including meropenem and ceftolozane–tazobactam, increased from 1.2 to 10.8% post-redefinition, despite isolates being wild-type and treatable with optimized *β*-lactam dosing [5]. In both settings, clinicians misinterpreted the new ‘I’ as ‘uncertain’ rather than SIE.

These findings highlight a critical misinterpretation by clinicians: changing a definition is not enough. Practices, habits and antimicrobial stewardship frameworks must change too. Notably, hospitals that implemented selective reporting, i.e. hiding meropenem susceptibility when a *β*-lactam was suitable at increased exposure, reduced inappropriate carbapenem use and reinforced proper interpretation of the SIE concept [6].

## *Enterobacterales* and the rise of dose-dependent logic

Breakpoint changes in *Enterobacterales* species have similarly affected drug categorization and prescribing. For cefuroxime, a commonly prescribed cephalosporin for urinary tract infections (UTIs), revised EUCAST breakpoints introduced ‘I’ as the predominant or often sole susceptibility category for *Escherichia coli* isolates. In a paediatric nephrology cohort in Poland, over a third of *E. coli* UTI isolates were categorized as ‘I’ to cefuroxime. This prompted a shift in treatment practices, with higher doses of cefuroxime used in preference to escalation, thereby demonstrating the utility of the SIE model in maintaining the clinical utility of older, narrower-spectrum drugs through dose optimization [7].

Such reinterpretation is also relevant for intravenous *β*-lactams. Clinical trials in AmpC-producing *Enterobacterales* bloodstream infections report that appropriately dosed cefepime is non-inferior to carbapenems. These findings have been incorporated into European Society of Clinical Microbiology and Infectious Diseases guidance, promoting high-dose cefepime as a viable option and illustrating how the ‘I’ category can guide rational carbapenem-sparing strategies [8].

## A system still catching up

Despite these advances, challenges remain. Surveillance systems frequently aggregate ‘I’ and ‘R’ results, misrepresenting true resistance rates and masking the potential clinical utility of dose-optimized agents. For example, the Canadian Integrated Program for Antimicrobial Resistance Surveillance programme reports ciprofloxacin susceptibility only as ‘susceptible’ or ‘non-susceptible (I/R)’, eliminating any distinction between agents treatable with SIE and those truly resistant [9]. This approach is mirrored in global surveillance platforms such as ResistanceMap, where ‘intermediate’ and resistance data are combined in reported metrics [10]. The same issue extends to published studies, where intermediate and resistant categories are often combined for simplicity or due to perceived clinical equivalence, further skewing epidemiological trends [2].

At the point of care, electronic prescribing systems and decision-support tools frequently flag ‘I’ agents as suboptimal, reinforcing interpretations that equate ‘intermediate’ with treatment failure, despite the original intent of the category to signal that efficacy is achievable with higher dosing or in drug-concentrating sites such as the urinary tract. Many hospital antibiogram tools apply colour-coded alerts, such as green for susceptible, yellow for intermediate and red for resistant, which may unintentionally encourage escalation when ‘yellow’ appears, even in cases where pharmacokinetic optimization would suffice [6].

The reinterpretation of ‘I’ as SIE is not a mere semantic change. It has exposed structural limitations in how we generate and apply evidence. Many clinical trials conducted under older EUCAST criteria excluded ‘intermediate’ isolates from analysis or grouped them with ‘resistant’ strains, a practice that now limits the relevance of their conclusions in the context of revised breakpoints. Guidelines, too, often retain recommendations shaped by S/I/R categorizations that failed to consider dose-exposure dynamics, while surveillance networks continue to report ‘resistance’ in ways that overlook recent advances in susceptibility interpretation [2]. These disparities risk sustaining outdated infection management, as much of the legacy evidence base remains misaligned with the revised ‘I’ interpretation, necessitating guideline and surveillance updates to integrate current antimicrobial susceptibility reporting into clinical practice.

## Conclusion: the weight of a letter

The reinterpretation of ‘I’ compels a revisit of our evidence base with clearer eyes and higher standards. It invites us to question how many of our practices are relics of flawed susceptibility classifications. And it reminds us that even a single letter, when misinterpreted or misapplied, can shape decades of prescribing behaviour, antimicrobial stewardship policy and patient outcomes.

## Abbreviations

EUCAST: European Committee on Antimicrobial Susceptibility Testing
PK/PD: pharmacokinetic/pharmacodynamic
SIE: susceptible, increased exposure
UTI: urinary tract infection
